# Large-Scale Virtual Screening Against the MET Kinase Domain Identifies a New Putative Inhibitor Type

**DOI:** 10.3390/molecules25040938

**Published:** 2020-02-19

**Authors:** Emmanuel Bresso, Alessandro Furlan, Philippe Noel, Vincent Leroux, Flavio Maina, Rosanna Dono, Bernard Maigret

**Affiliations:** 1 Université de Lorraine, CNRS, Inria, LORIA, F-54000 Nancy, France; 2 Aix Marseille Univ, CNRS, Developmental Biology Institute of Marseille (IBDM), UMR7288, Parc Scientifique de Luminy, 13009 Marseille, France; 3 University of Lille, CNRS, UMR 8523, PhLAM-Physique des Lasers Atomes et Molécules, F-59000 Lille, France

**Keywords:** virtual screening, ensemble-docking, structure-based drug design, cross-docking validation, induced fit, conformational selection, MET kinase

## Abstract

By using an ensemble-docking strategy, we undertook a large-scale virtual screening campaign in order to identify new putative hits against the MET kinase target. Following a large molecular dynamics sampling of its conformational space, a set of 45 conformers of the kinase was retained as docking targets to take into account the flexibility of the binding site moieties. Our screening funnel started from about 80,000 chemical compounds to be tested in silico for their potential affinities towards the kinase binding site. The top 100 molecules selected—thanks to the molecular docking results—were further analyzed for their interactions, and 25 of the most promising ligands were tested for their ability to inhibit MET activity in cells. F0514-4011 compound was the most efficient and impaired this scattering response to HGF (Hepatocyte Growth Factor) with an IC${}_{50}$ of 7.2 μM. Interestingly, careful docking analysis of this molecule with MET suggests a possible conformation halfway between classical type-I and type-II MET inhibitors, with an additional region of interaction. This compound could therefore be an innovative seed to be repositioned from its initial antiviral purpose towards the field of MET inhibitors. Altogether, these results validate our ensemble docking strategy as a cost-effective functional method for drug development.

## 1. Introduction

MET receptor is a multifunctional receptor tyrosine kinase that plays a pivotal role in human development and tumorigenesis. Upon binding of its ligand HGF (Hepatocyte Growth Factor), MET triggers several intracellular signaling cascades, including MAPK and PI3K pathways, leading to various cellular responses, such as survival, proliferation, and migration, among others. MET activation can be driven in cancer by several mechanisms: HGF or MET overexpression, and also mutations [1].

Aberrant activation of MET signaling does not only affect cancer development and progression, but it also contributes to resistance against other cancer drugs [2,3,4,5,6,7,8,9,10,11]. Consequently, MET represents a pharmaceutically relevant target in anticancer drug design and has been the focus of several anticancer strategies [12,13,14,15,16,17,18]. Pioneer MET inhibitors such as SU11274, PHA665752, or AM7 were helpful for demonstrating the efficacy of MET inhibition to impair tumor growth in preclinical models. Then, further developments in the field led to the approval by the FDA of crizotinib and cabozantinib in the 2010s for treating non-small cell lung cancers and medullary thyroid cancers, respectively.

Even though promising results have been reported, the therapeutic activity of these drugs is still relative and efforts are required to identify new MET inhibitors with physicochemical properties optimized for clinical efficiency [19,20]. Moreover, new alterations in MET sequence have been recently identified, such as MET exon 14 skipping in lung cancers and the emergence of MET mutations in the kinase domain following treatment with MET inhibitors [21]. Novel inhibitor structures may possibly target these mutations with increased efficiency.

Designing new putative hits against MET therefore remains a valuable challenge to be tackled. In the present work, we carried out a virtual screening campaign in order to identify innovative compounds able to become new hits for further lead development. As MET plasticity upon ligand binding had been previously highlighted [22,23], we took into account this aspect for the molecular docking simulations. Indeed, MET can accommodate several distinct ligand binding modes and associated receptor conformations, a feature that is particular to the kinase family [24]. We reasoned that it should be taken into account for designing drugs with improved efficiency and selectivity profiles [25,26]. To be efficient, the molecular docking engines embedded within the virtual screening approaches must be adapted to handle such flexibility [27,28]. In the present work, we used the ensemble-docking strategy—previously recognized for its efficiency in drug design [29]—and show the benefit of an investigation using a large ensemble-docking on MET.

In previous medicinal chemistry works, we already identified novel MET inhibitors [30,31] and characterized the different MET ligand binding modes as shown by the stream of released X-ray data [32]. Here, we provide the results of a large-scale ensemble-docking investigation on MET, in which MET conformations are extended from available X-ray data to molecular dynamics and normal mode analysis. A limited number of candidate compounds were selected from the ensemble-docking results and one of them was subsequently validated experimentally as a possible new MET inhibitor, providing a valuable seed for further investigations.

## 2. Materials and Methods

### 2.1. Screened Chemical Libraries

The choice of an appropriated set of compounds to explore the virtual screening space is critical for assuming a good rate of success [33]. Today, millions of compounds can be selected for high-throughput screening, and a suitable selection strategy must be designed. In our case and according to previous success stories [34,35,36], we chose to use a set of libraries selected in order:

1. To use the highest possible chemical diversity, in order to cover a large spectrum of chemical structures [37,38,39,40];
2. To include kinase-targeted compounds, as such a choice is already proven to be successful [41,42];
3. To explore natural products, which are a promising source of anticancer drugs [43,44,45];
4. To take into account the repositioning of approved compounds, as drug repurposing presents an increasing interest in cancer research, by removing many costs associated with several steps of drug development [46,47,48,49]. Several proofs of concept are now available and a typical case of viral-to-cancer drug repositioning is gemcitabine with US patent No 4,808,614, aimed at treating viral infections, and the later-issued US No 5,464,826, which claims of its use to treat cancer. Therefore, we also considered chemical libraries dedicated to antiviral compounds.

According to the criteria listed above concerning the choice of the chemical libraries, we firstly downloaded around 200,000 compounds from the chemical providers listed in Table 1. After eliminating redundancies in compounds and in scaffolds to assume a large chemical diversity and in respect of general druglike definitions [50], we finally retained about 80,000 compounds for our screening campaign.

### 2.2. Selected MET Conformational Ensemble

In ensemble-based docking calculations, a well-suited choice of the protein target conformational sample is required to reproduce the protein plasticity and the possible induced-fit phenomena [51,52].

**Figure 1:**
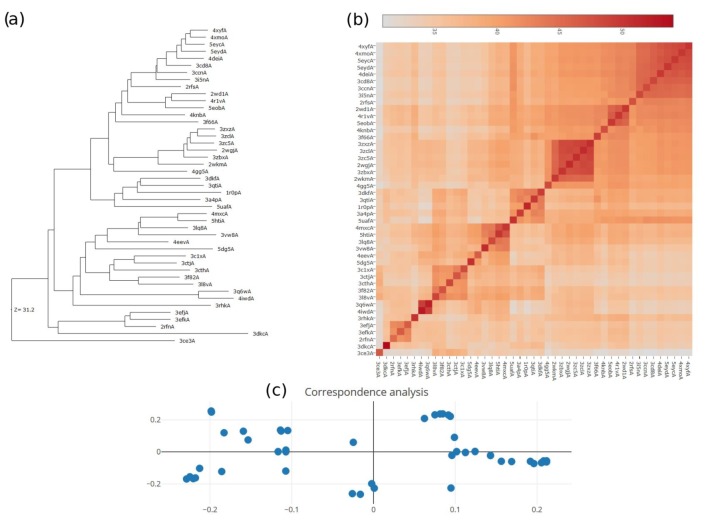
(**a**) Dendrogram showing the relationships between the 45 PDB conformers listed in Table 2 and Table 3 and used to sample MET structure plasticity. (**b**) Similarity heat-map showing the relationships between the 45 PDB conformers and used to sample MET structure plasticity. The color scale corresponds to the Dali Z-score values. (**c**) Correspondence analysis of the 45 ensemble PDB-related conformers. This plot positions data points with the most similar structural neighborhoods near each other according to a multidimensional scaling method.

Concerning MET conformational flexibility, our previous exploration by molecular dynamics and normal mode simulations [22] was limited to the 26 PDB structures available at that time (Table 2). We have now extended this analysis by considering all the X-ray structures available for the wild-type MET in the PDB [53] deposited after 2012. From the additional structures found, only 19 were considered in this work (Table 3) as we discarded those where three regions were missing in the X-ray structure and those where the number of missing residues in a single region was larger than 20. This selection aimed to improve our protein ensemble sample by covering most of the kinase structural characteristics, such as the position of the c-helix (in or out) or of the aspartate-phenylalanine-glycine (DFG) motif (in or out) as defined in Kinametrix [54], thus covering most of the inhibitor type binding modes. 3D structures considered in this ensemble of 45 conformers looked representative of the diversity of MET structures, as shown from the dendrogram, the heat maps, and correspondence maps in Figure 1. These results obtained thanks to the Dali server [55] clearly illustrate how MET 3D structures used here are organized into several families covering most of the protein conformational space presently known.

To be in accordance with the 26 conformers coming from our previous work [22], the 19 added crystal structures were prepared and cleaned following the same protocol: missing residues, side chains, and hydrogens were added when necessary; unnecessary water molecules, ions, and additives were removed; basic and acidic side chains were ionized according to a pH set to 7. To consider possible binding sites fluctuations, short molecular dynamics (MD) simulations were undertaken for each of these 19 structures. For that purpose, these structures were placed in a solvent box of 80 Å and counter ions were added for electrostatic neutrality. NAMD [57] molecular dynamics program was used with the same CHARMM36 force field and same protocol as previously described [22]. After minimization and equilibrium steps (64,000 conjugate gradients and 1 ns MD, respectively), 10 ns of MD were recorded, with a frame length of 1 ps. These 19 MD trajectories were analyzed, and the most stable representative conformer was retained for each of them and added in the ensemble-docking set.

### 2.3. Description of the Ensemble-Docking Protocol

The ensemble-docking facility proposed in the GOLD docking program was used [58]. This GOLD feature evaluates different receptor conformations concurrently during the docking exploration. The protein ensemble used in this work thus contained 45 MET conformers (26 from our previous work and 19 added in this one). As these conformers must be superimposed before being used in GOLD ensemble-docking program, they were structurally aligned according to their conserved and most rigid secondary structure patterns, as previously described [22] and summarized in Table 4.

**Figure 2:**
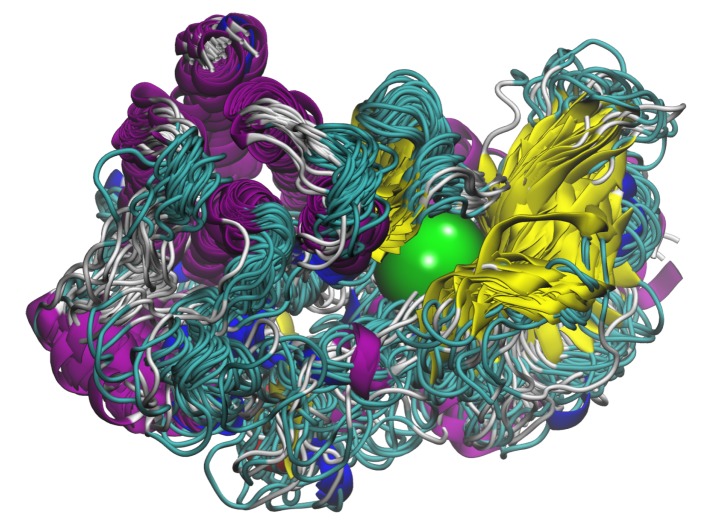
Position of the average center-point (as a green sphere) found from the 45 used conformers and used for the ensemble-dockings.

When docking an ensemble of conformations for a given protein, their binding sites must be defined using a method that is not conformer specific. In the present ensemble-GOLD version, as it was not possible to define the active site by a list of atoms or residues, the only way was to use the centroid of the binding cavity and a sphere radius around this point. Therefore, for each of the 45 aligned protein conformers used here, protein cavities and their center of mass were detected by the LIGSITE program [59]. From these data, we obtained an average center point as the ensemble binding site definition for GOLD. Figure 2 presents the position of this average center point within the 45 protein conformers. A radius of 20 Å was associated to this average point to define the binding cavity of each conformer in order to correctly encompass the receptor for all the conformations in the ensemble, including conformational variations around the center. We also verified that the resulting sphere was encompassing all groups of residues previously identified as potential interaction areas for MET ligands [32].

For each docking run, we used 50 starting poses/molecule for the GOLD generic algorithm. Tested compounds were ranked by the standard goldscore scoring function.

### 2.4. Computer Grid Facilities

Due to the massive calculations needed ( 80,000 molecules × 48 protein ensemble conformers × 50 poses/molecule), and considering the computing time to achieve only one run, we used the Grid5000 facility [60] providing the required computer power in order to distribute the calculations using the PVM framework embedded in GOLD. A total of 1300 cpus (mostly Xeons) with 4 GB RAM/core and infiniband connections were used for each run. The docking performances run around 300-docked ligands/ensemble/hour. The calculations were spread on the clusters using the same strategy as previously described [61].

### 2.5. Scattering Assays

The experimental protocols for measuring the potency of MET inhibitors are detailed in previous publications [30,62]. MDCK cells were preincubated with compounds overnight at 0.1–100 μM concentrations at 37 °C in a humidified atmosphere of 5% CO${}_{2}$, followed by a 24 h stimulation with 20 ng/mL HGF (R&D Systems). Cells were further incubated at 37 °C in an atmosphere of 5% CO${}_{2}$ for 24–48 h, washed with phosphate buffered saline (PBS; Gibco BRL), and fixed with 4% PFA (paraformaldehyde, Sigma). The quantification of scattering response was performed by counting the number of cells with scattered morphology in 30 independent colonies. The IC${}_{50}$ corresponds to the concentration of compounds leading to a 50% inhibition of MET-triggered cell scattering.

## 3. Results

### 3.1. Preliminary Validation Concerning the GOLD Ensemble-Docking Protocol Used

The coordinates of the 45 aligned conformers and of the sphere representing their common binding sites constituted our ensemble-docking protein reference.

**Figure 3:**
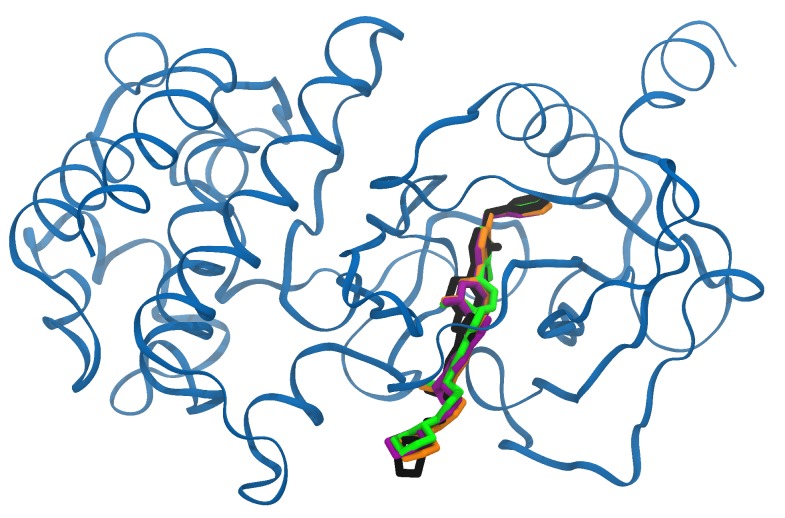
Poses of the AM7 ligand in the X-ray 2RFN structure compared to the docking results. In black, the original pose of the ligand in its PDB protein conformation; in colors, the best docking poses obtained by GOLD on the 2RFN target using a definition of the binding site from a list of residues (orange), from the original ligand (green), and from a center-point (purple).

The first question here concerned the accuracy of this binding site definition compared to ones that are more classical. For that, we compared the docking results for some of the selected 45 MET conformers using three binding site definitions; namely, a residue list, an existing ligand, and the center point of the binding cavity, respectively. For each individual docking target, the three definitions provided almost the same rank and docking score for the associated PDB ligand (Table 5). Moreover, the poses of this ligand found using the three binding site definitions were similar to the pose found in the crystal structures, as illustrated with the example of the AM7 ligand on Figure 3.

The second question was related to the ability of the ensemble-docking process to retrieve a given PDB ligand to its PDB structure among the 45 ones. To evaluate that point, an ensemble-docking calculation was carried out on the 45 protein target conformers using a short chemical library built from their own 45 associated ligands (the list is given in Table 2 and Table 3). We checked whether we could associate the right PDB target for a given PDB ligand (with possibly similar rank, score and pose compared to the ones found for the individual target dockings) in the protein ensemble. This was achieved for almost 80% of the compounds (Table S1). For example, the KSA ligand was able to preferentially retrieve its original 1R0P partner among the ensemble of the 45 PDB conformers of the protein target.

From these results, it appeared that the ensemble-docking procedure we used was a satisfactory method to tackle multiple conformers docking and to achieve a valuable virtual screening.

### 3.2. Selection of Candidate Hits from the Virtual Screening Campaign

Once the screening campaign was achieved for the 80,000 compounds filtered from the chosen libraries, we kept the top-100 ranked compounds according to their GOLD scores (ranging from 100 to 114) for further analysis.

We started the docking analysis with the Life Chemicals compound F0725-0356 giving the best docking score of 114. A comparison between the X-ray complex 3EFK/MT4 structure and the MD_3EFK/F025-0356 one presented quite similar poses and protein/ligand interactions. Indeed, the most important residues known in MET interactions (namely, Met${}_{1160}$, Asp${}_{1222}$, Tyr${}_{1159}$) were found in both complexes.

We next analyzed the protein-ligand interactions for the other top-100 compounds in order to compare them to the ones found in the 45 original PDB structures (Table 2 and Table 3). For that, we used the PLIP program [63] by focusing on two important interaction types: hydrogen bonds and π-stacking. Protein residues Met${}_{1160}$ (45/45), Asp${}_{1222}$ (34/45), and Lys${}_{1110}$ (6/45) concentrated the vast majority of hydrogen bonds with ligands; while Tyr${}_{1230}$ (25/45) and Phe${}_{1223}$ (7/45) dealt with most of the π-stacking. In order to limit our biological tests on possible promising compounds, we eliminated from the top-100 list the molecules not presenting at least one hydrogen bond and one π-stacking from the ones described above in the PDB complexes.

After this filter, we retained only 41 compounds as satisfying these criteria. As most of these compounds came from the Life Chemical antiviral library and given the simplicity of comparing molecules from the same supplier, we decided to only test compounds from Life Chemicals. As some of these molecules were not available in stock from this provider, only the 25 compounds listed in Table 6 were finally kept to proceed further.

### 3.3. F0539-1482 and F0514-4011 Inhibit MET-Induced Cell Scattering

These 25 compounds were then experimentally tested for their ability to restrain MET-triggered biological activities. We previously efficiently screened compounds for their inhibitory properties towards MET-triggered biological responses by using cell scattering assays [31,64]. In particular, MDCK epithelial cells acquire a “scattered phenotype” after stimulation with MET ligand HGF.

**Figure 4:**
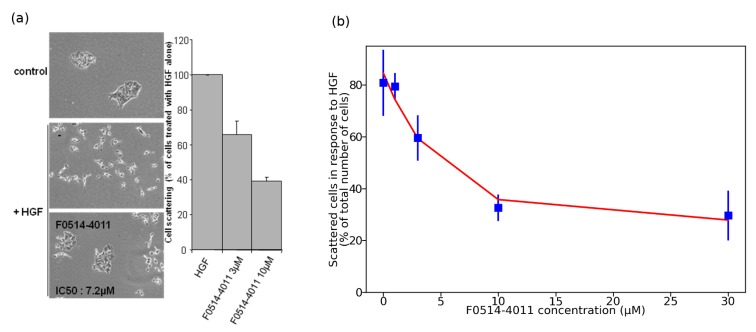
(**a**) F0514-4011 impairs cell scattering in response to MET ligand HGF: MDCK epithelial cells were treated with 20 ng/mL HGF, with or without preincubation with F0514-4011 for 2 h. F0514-4011 IC${}_{50}$ is 7.2 μM. (**b**) Dose-response curve for F0514-4011.

Among the 25 tested compounds, two compounds were found active, namely, F0539-1482 and F0514-4011. F0514-4011 was the most efficient and impaired this scattering response to HGF with an IC${}_{50}$ of 7.2 μM (Figure 4). No toxic effects were observed at biologically active concentrations. This study thus demonstrates that our strategy actually allows the identification of compounds able to inhibit MET-driven biological activities.

### 3.4. Compared Docking of F0514-4011 Compound Versus Known Inhibitors

**Figure 5:**
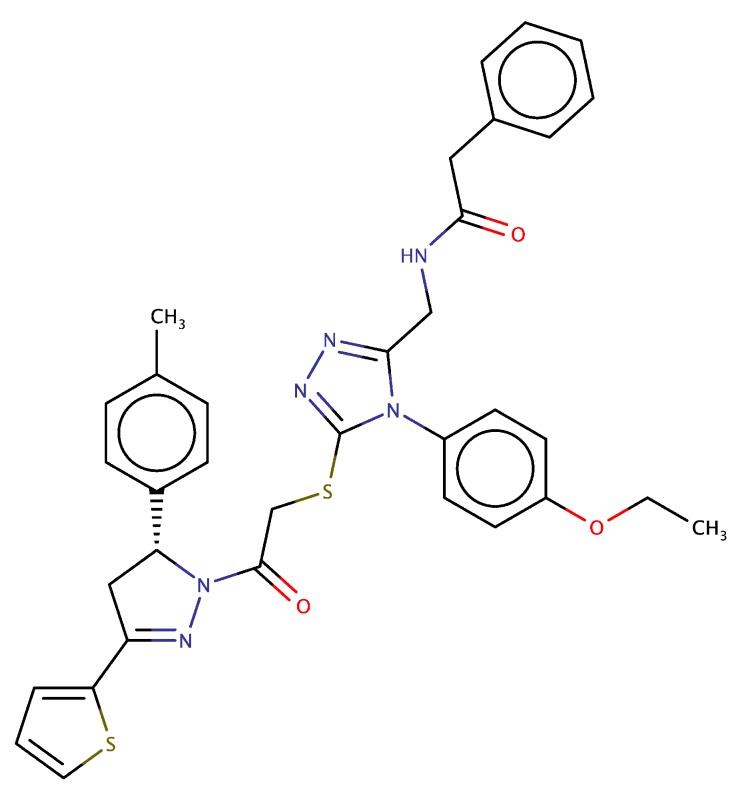
F0514-4011: N-[[4-(4-Ethoxyphenyl)-5-[2-[3-(4-methylphenyl)-5-thiophen-2-yl-3,4-dihydropyrazol -2-yl]-2-oxoethyl]sulfanyl-1,2,4-triazol-3-yl]methyl]-2-phenylacetamide.

In order to understand why the compound F0514-4011 (Figure 5) was the most potent compound among the 25 experimentally tested ones while not presenting the highest GOLD score, we compared its docking data with those of potent existing inhibitors. For that, we collected the structures of ligands found in the PDB related to MET kinase domain in complex with already marketed inhibitors with binding IC${}_{50}$ found in the nM range (Table 7). All these compounds were submitted to the ensemble-docking GOLD protocol already used for our virtual screening campaign. From these calculations, it appeared that the best docking scores ranged from 111 for merestinib (L1X ID in PDB 4EEV) to 83 for AMG337 (5T1 ID in PDB 5EYD), so that the score of 103 obtained for our active F0514-4011 compound was in this range of active compounds. Considering now IC${}_{50}$, one possibility to explain the higher IC${}_{50}$ of 7.2 μM obtained for F0514-4011 (compared to 0.4–14 μM range found for compounds listed in Table 7) could be its weaker solubility (cLogP of 5.7, greater than that of all compounds listed in Table 7).

**Figure 6:**
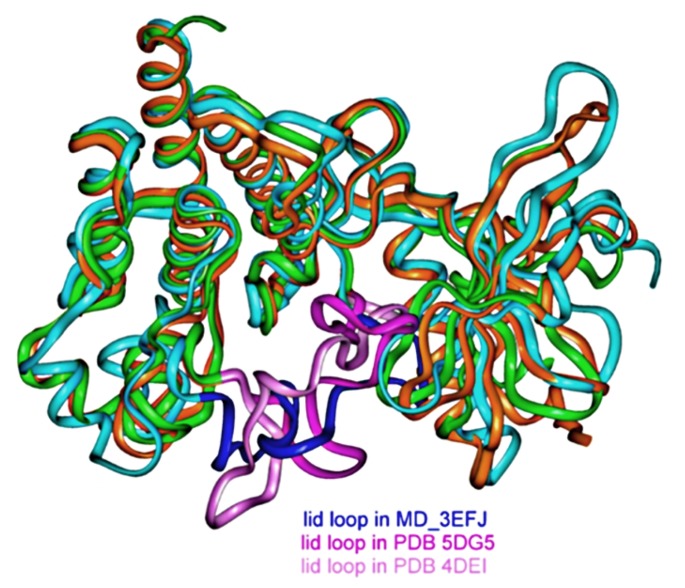
Differences for the lid DFG loop between selected PDB structures and our MD-refined MD_3EFJ conformations. The proteins are depicted from their C${}^{\alpha }$ ribbon-like traces.

Another point concerned the interaction of F0514-4011 with amino acid residues within the protein-binding region. In Table 8, we have listed the protein residues/ligand interactions found from Table 7 PDB complexes, as calculated by the PLIP program. These interactions were compared to the ones obtained for F0514-4011 from its best pose MD_3EFJ in the ensemble MET conformations. From this comparison, it appears that several of the most important amino acid residues found from the PDB protein/ligand analysis were also found for F0514-4011, at the exception of Met${}_{1160}$, common to all PDB structures of Table 8, replaced possibly by Met${}_{1131}$ and Met${}_{1229}$ in our case. This situation is mostly due to the conformation of the large DFG loop acting as a highly flexible lid protecting the binding sites which was quite different in the MD_3EFJ conformation, found as the most suitable one to bind F0514-4011 when compared to the PDB ones (see Figure 6 for an example with the 5DG5 and 4DEI structures). Therefore, our docking results concerning the best pose proposed by GOLD for F0514-4011 appear quite in agreement with most of data obtained from all the PDB concerning MET kinase domain complexed with inhibitors.

**Figure 7:**
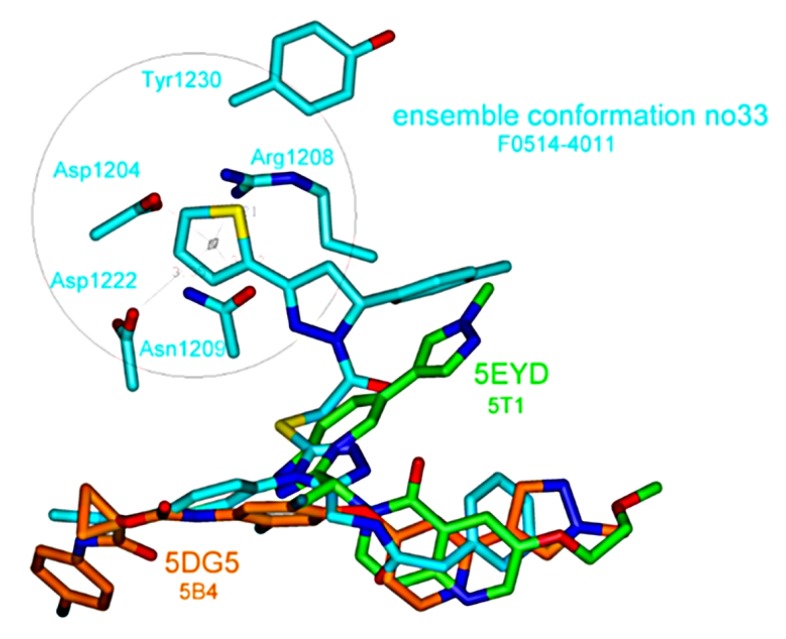
Comparison of the conformations between F0514-4011, the U-shape inhibitor 5T1 (AMG337), and the linear-shape 5B4 (altiratinib), as observed in their respective binding sites.

To further characterize the F0514-4011 inhibitor type, we have considered the general 3D shape of known kinase inhibitors as analyzed in several papers [7,65,66,67]. Concerning MET, such compounds are generally classified as type-I or -II. Type-I ligands essentially bind at the ATP binding site and present a U-shaped conformation, with the protein in the DFG-in structure; while type II are found in an extended shape and correspond to the DFG-out protein form. We illustrate this in Figure 7, showing the conformations of two typical ligands, namely, type-I AMG337 (from PDB 5EYD) and the type-II altiratinin analog DP-4157 (from PDB 5DG5). From this picture, it appears that F0514-4011 presents both the U and linear shapes while also showing another region of interaction, including three of the protein residues already found in MET complexes—namely, Asp${}_{1222}$, Tyr${}_{1230}$, and Arg${}_{1208}$ (found only 2 times for 3C1X and 3YW8 among our 45 ensemble conformations). Asp${}_{1204}$ and Asn${}_{1209}$ residues, still not involved in MET complex PDB structures, complement this supplementary binding pocket. The thiophene moiety of F0514-4001 was placed central within this pocket by the thiophene-pyrazole group which also oriented the associated toluene ring to close the U-shape part. Therefore, one could postulate that F0514-4011 molecule describes a possibly novel type of inhibitor.

**Figure 8:**
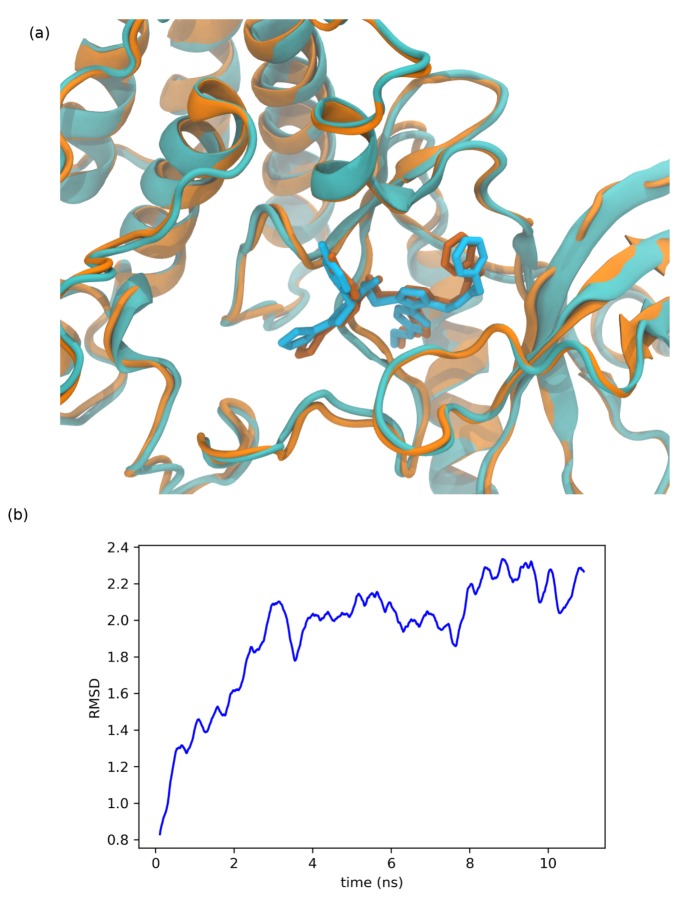
(**a**) Comparison between the initial docking pose (in orange) of F0514-4011 and the final one after the 10-ns MD simulation (in cyan). (**b**) Evolution of F0514-4011 root mean square deviation (RMSD) during 11 ns (1 ns equilibrium and 10 ns production) of MD simulation. Poses were aligned on the initial one and the curve was smoothed.

Nevertheless, considering the limitations of any docking program, the stability of F0514-4011’s best docking pose could be questioned. In order to validate it, we have performed a molecular dynamics simulation using the same conditions as those used for the PDB complexes (cubic water box of 80 Å^3^). The results show that the GOLD docking pose is very stable and still conserved after 10 ns of MD (Figure 8). The protein/ligand interactions found for F0514-4011 after the MD simulation were similar to those discussed above, thus giving confidence to the robustness of the docking results. Our final question concerns the originality of F0514-4011 compared to the known MET ligands. The Tanimoto similarity index calculated between F0514-4011 and most of the published MET ligands shows that the molecule identified by our virtual screening campaign seems to be an innovative hit as all the Tanimoto values are low, ranging from 0.39 (with the pioneer inhibitor PHA-665752) to 0.12 (for norcantharidin) (Table S2). We have completed this quite elementary similarity search by using the ChemDes web server [68], which allows a large panel of similarity fingerprint types as well as fingerprints descriptors and similarity measures. Using this web server, we mined several databases collecting MET known inhibitors (such as the PDB or PubChem [69]), already in clinical trials (such as MDDR [70]), or described as putative inhibitors (such as in Life Chemical or sellekchem [71] providers). The results obtained with this method confirmed the lack of similarity suggested with the Tanimoto distance. With the Sokal similarity method and DTRF fingerprint types, the similarities ranged from 0.46 to 0.19 (in the PDB list, a maximum of 0.40 was obtained for compound ID 75H found in PDB ID 5T3Q (data not shown)).This could be due to the thiophene moiety of F0514-4011, as we have found only two papers and one patent in the literature referring to thiophene-related MET inhibitors [72,73,74] and only one reference to the role of thiophene-pyrazole moiety in kinase inhibition [75].

## 4. Discussion

Molecular docking, molecular dynamics, and virtual screening approaches can now be efficiently used for the design of new inhibitors of the MET kinase domain [27,56,76,77,78,79,80]. From all these approaches, new potent compounds were obtained and more highlights revealed about MET kinase domain conformational behavior. In this vein, our study merges both simulations and experiments and highlights a novel scaffold for MET inhibition.

Using an ensemble-docking approach associated to short molecular dynamics runs in order to take into account the flexibility of the used X-ray structures in the protein conformational ensemble, we were faced with the fundamental question of the relevance of this strategy for handling the difficult problem of predicting ligand-binding modes on a flexible target. This is especially true for MET kinase, the active site of which exhibits important structural variations, as observed in their available crystal structures [81,82]. We believe that this work brings a positive answer to this question and can constitute a working line for other simulations in the future. Ensemble-docking is now widely used, and incorporating this approach to short molecular dynamics simulations looks promising. Still, a couple of simple questions have to be answered prior to initiating the docking calculation: how do we generate a relevant ensemble for a given receptor [51], and how can we be sure that the possible energy differences obtained between conformations in the ensemble are properly accounted for?

Interestingly, F0514-4011 compound (also referenced in PubChem with ID 5237313) is not a newcomer in drug design as it has been already tested as a possible activator of E3 ligase (FBW7) and inhibitor of microphthalmia-associated transcription factor (MITF), but was found inactive in both assays. Our study suggests that it could be repositioned for MET inhibition, as evidenced by its biological activity against MET-driven cell scattering. Some drug properties such as solubility and lack of toxicity were already known. With regard to its molecular weight of 650Da, which could be considered as limiting its possible therapeutic action, it should be noted that other inhibitors currently on the market have similar characteristics such as tarloxatinib (679Da), foretinib (632Da), or golvatinib (633Da). Therefore, it should not be a major hurdle if lead optimization provides us with a promising drug in terms of activity and/or selectivity. This will be the topic of future investigations.

This virtual screening work presents F0414-4011 as a valuable compound that could be a seed for developing new and innovative leads against MET kinase. Its novelty and originality might be used to overcome the resistance problem found presently for several existing inhibitors.

## Figures and Tables

**Table 1:** List of the selected chemical libraries used in the present virtual screening campaign, providing a total of 76,251 compounds.

Supplier	Library Names
French laboratories chimiotheque-nationale.enscm.fr	Selected subsets (kinase, essential, etc.)
Collaboration medchem.u-strasbg.fr	laboratory collection
ChemBridge www.chembridge.com/index.php	kinase library diversity library
Life Chemicals www.lifechemicals.com	kinase general library antiviral library
	15K diversity library
TimTec www.timtec.net	NDL + NPL natural derivatives library
Otava otavachemicals.com	all kinase library
	10K diversity library
	natural productlike library
TargetMol www.targetmol.com	antivirus library natural compounds library

**Table 2:** List of the 26 PDB MET kinase domain structures selected in the previous work [22] and reused in the present one. The kinase conformation associated to each structure is annotated according to the KinaMetrix web resource [56]: DO means DFG-out, DI means DFG-in, CO means α-cHelix-out, CI means α-cHelix-in, and ωCD indicates DFG intermediate.

N^*o*^	PDB ID	Ligand PDB ID	Deposition Date	Annotation
1	1R0P	KSA	2003	Inactive CODI
2	2RFN	AM7	2007	?
3	2RFS	AM8	2007	Inactive CODI
4	3C1X	CKK	2008	Inactive CIDO
5	3CCN	LKG	2008	Inactive CODI
6	3CD8	L5G	2008	Inactive CODI
7	3CE3	1FN	2008	Inactive CODO
8	3CTH	319	2008	Inactive CIDO
9	3CTJ	320	2008	Inactive CIDO
10	3F66	IHX	2008	Inactive CODI
11	3F82	353	2008	Inactive CIDO
12	3EFJ	MT3	2008	Inactive CODO
13	3EFK	MT4	2008	Inactive CODI
14	2WD1	ZZY	2009	Inactive CODI
15	3DKC	ATP	2009	Inactive CODI
16	3DKF	SX8	2009	Inactive CODI
17	2WGJ	VGH	2009	Active CIDI
18	3A4P	DFQ	2010	Inactive CODI
19	3L8V	L8V	2010	Inactive CIDO
20	3I5N	B2D	2010	Inactive CODI
21	3LQ8	88Z	2010	Inactive CODO
22	2WKM	PFY	2010	Inactive CODI
23	3Q6W	Q6W	2011	Active CIDI
24	3QTI	3QT	2011	Inactive CODI
25	3RHK	M97	2011	Inactive ωCD
26	3ZXZ	KRW	2011	Inactive CODI

**Table 3:** List of the PDB MET kinase domain structures added to the ones coming from our previous work [22]. The kinase conformation associated to each structure is annotated according to the KinaMetrix web resource [56]: DO means DFG-out, DI means DFG-in, CO means α-cHelix-out, CI means α-cHelix-in, and ωCD indicates DFG intermediate.

N^*o*^	PDB ID	Ligand ID	Ligand IC${}_{50}$ (nM)	Date	Missing Sequence	# Missing Residues	Annotation
1	4DEI	0JL	0.6–2	2012	1100–1103 1115–1117	7	Inactive CODI
2	4GG5	0J3	0.9	2012	1146–1151	6	Inactive CODI
3	4EEV	L1X	4.7/42	2012	1225–1243	19	Inactive CIDO
4	3VW8	DF6	2	2013	1237–1242 1286–1290	11	?
5	4IWD	1JC	1	2013	1234–1235 1240–1243	6	Active CIDI
6	3ZCL	5TF	19	2013	1100–1102	3	Inactive CODI
7	3ZC5	W9Z	6	2013	1099–1102	4	Inactive CODI
8	3ZBX	6XE	5	2013	1089–1102	14	Inactive CODI
9	4KNB	1RU	47/410	2013	1099–1103 1113–1115	8	Inactive CODI
10	4MXC	DWF	6.7	2014	1238–1242	5	Inactive CODO
11	4XYF	44X	1/5	2015	1099–1103	5	
12	4R1V	3E8	400	2015	1150–1151	2	Inactive CODI
13	4XMO	46G	2	2015	1098–1103	6	Inactive CODI
14	5DG5	5B4	?	2015	-	-	Inactive CODO
15	5EYD	5T1	1	2016	1098–1103 1151–1152	8	Inactive CODI
16	5EOB	5QQ	0.24	2016	1238–1240	3	Inactive CODI
17	5EYC	5SZ	3	2016	1099–1103	5	Inactive CODI
18	5UAF	84P	?	2017	1098–1105 1145–1152	16	Active CIDI
19	5HTI	66L	?	2017	1238–1242	5	Inactive CODO

**Table 4:** List of the secondary structure elements used for aligning all the conformers.

Domain	Secondary Structure Name	Residues
N-terminal	β1	1076 to 1081
	β2	1092 to 1098
	β2	1104 to 1110
	β2	1144 to 1146
	β2	1154 to 1158
C-terminal	αE	1178 to 1198
	αF	1263 to 1278
	αH	1310 to 1320
	αI	1330 to 1343

**Table 5:** Comparison of the docking results using the 3 binding site definitions.

Definition of the Binding Site	Target PDB Name	Ligand PDB Name	Rank Number	Score Value
Center + radius 20 Å	3DKC	ATP	1	105.5
	2RFN	AM7	1	100.8
Residues list	3DKC	ATP	1	102.8
	2RFN	AM7	1	98.0
From its PDB ligand	3DKC	ATP	1	107.1
	2RFN	AM7	1	106.6

**Table 6:** Ligands selected from the Life Chemical (LC) antiviral library and experimentally tested. “-”: the compound (assessed at a concentration up to 100 μM) did not affect MDCK cell scattering in response to HGF/SF. “+”: the compound impaired MDCK cell scattering in response to HGF/SF with an IC${}_{50}$ > 10 μM. “+++”: the compound impaired MDCK cell scattering in response to HGF/SF with an IC${}_{50}$ < 10 μM.

Mol ID	Life Chemicals Name	GOLD Score	Best Protein Conformer	Biological Activity
1	F0725-0356	120.7	MD_3EFK	-
2	F0772-0607	111.8	MD_3EFK	-
3	F0816-0342	111.2	MD_3F82	-
4	F0737-0405	110.6	MD_3EFJ	-
5	F0737-0393	110.1	MD_3EFK	-
6	F0301-0263	105.5	MD_3EFK	-
7	F0721-0868	105.1	MD_3EFK	-
8	F0715-0299	105.0	MD_3EFK	-
9	F0539-1482	104.0	MD_3EFJ	+
10	F0385-0029	103.8	MD_3EFK	-
11	F0385-0334	103.4	MD_3EFJ	-
12	F0514-4011	103.3	MD_3EFJ	+++
13	F0174-0048	102.4	MD_3EFK	-
14	F1620-0074	102.1	MD_3EFJ	-
15	F0011-0324	102.0	MD_3EFK	-
16	F0721-0906	102.0	MD_3EFJ	-
17	F0012-0227	101.9	MD_3EFJ	-
18	F0721-0911	101.9	MD_3EFJ	-
19	F0715-0300	101.8	MD_2RFN	-
20	F2252-0240	101.1	MD_3EFK	-
21	F0772-2099	100.9	MD_3EFJ	-
22	F0473-0261	100.9	MD_3CE3	-
23	F0721-0900	100.5	MD_3EFK	-
24	F0772-2147	100.5	MD_3EFJ	-
25	F0526-0094	100.3	MD_3EFJ	-

**Table 7:** Data used for some known marketed inhibitors with nano-molar range IC${}_{50}$ found in the PDB.

PDB ID	Ligand ID	Name	IC50	Solubility cLogP	Docking Score
2RFS	AM8	SU11274	10 nM	2.9	86
2WGJ	VGH	Criotinib	11 nM	3.5	82
2WKM	PFY	PHA-665752	9 nM	4.4	88
3DKF	SX8	SGX-523	4 nM	1.4	84
3RHK	M97	Tivantinib	4 nM	3.1	83
3LQ8	88Z	Fortinib	0.4 nM	4.3	99
3Q6W	Q6W	MK-2461	0.4 nM	3.3	93
3QTI	3QT	NVP-BVU972	14 nM	1.6	84
3ZXZ	KRW	PF-04217903	5 nM	0.2	87
4EEV	L1X	Merestinib	5 nM	3.4	111
5EYD	5T1	AMG337	1 nM	0.3	83

**Table 8:** List of the protein residues interacting with a nM. inhibitor from the PDB complexes of Table 7 ranked by their number of occurrence. In bold, the residues also found in the interactions with F0514-4011 with the MD-3EFJ MET conformation. According to the PLIP results, a residue was marked “+” when at least one protein-ligand interaction was found, whatever its quality (hydrophobic, H-bond, π-stacking, ionic, etc.) and marked by “-” when no protein-ligand interaction was found.

	PDB IDs										
Residue	4EEV	2WGJ	5EYD	3ZXZ	2RFS	3RHK	3QTI	3Q6W	2WKM	3DKF	3LQ8
MET${}_{1160}$	+	+	+	+	+	+	+	+	+	+	+
**LEU${}_{1157}$**	+	+	+	-	+	+	+	+	+	+	+
**ASP${}_{1222}$**	+	-	+	+	+	-	+	-	-	+	+
ALA${}_{1108}$	+	-	+	+	-	-	+	+	-	+	+
TYR${}_{1230}$	-	+	+	+	+	-	+	-	+	+	-
**VAL${}_{1092}$**	-	+	-	+	-	+	+	+	-	+	-
**ILE${}_{1084}$**	+	+	+	+		+	+	+	-	+	+
TYR${}_{1159}$	-	-	-	-	-	-	-	+	+	-	+
PRO${}_{1158}$	-	+	-	-	+	+	-	-	+	-	-
**LEU${}_{1140}$**	-	-	-	-	+	-	-	+	+	-	+
**ALA${}_{1221}$**	-	-	-	-	-	-	+	+	-	+	-
**PHE${}_{1223}$**	+	-	-	-	-	+	-	-	-	-	+
ASP${}_{1164}$	-	-	-	-	-	-	-	-	+	+	-
**LYS${}_{1110}$**	+	-	-	-	-	+	-	-	-	-	-
ASN${}_{1209}$	-	-	-	-	-	-	-	-	+	-	-
GLU${}_{1127}$	-	-	-	-	-	-	-	-	-	-	+
PHE${}_{1134}$	+	-	-	-	-	-	-	-	-	-	+
VAL${}_{1139}$	+	-	-	-	-	-	-	-	-	-	+
PHE${}_{1200}$	+	-	-	-	-	-	-	-	-	-	+
ARG${}_{1086}$	-	-	-	-	-	-	-	+	-	-	-
ARG${}_{1208}$	-	-	-	-	-	-	-	+	-	-	-
THR${}_{1343}$	-	-	-	-	-	-	+	-	-	-	-
GLU${}_{1347}$	-	-	-	-	-	-	+	-	-	-	-
**PHE${}_{1089}$**	-	-	-	-	-	+	-	-	-	-	-
ASP${}_{1231}$	-	-	-	+	-	-	-	-	-	-	-
ARG${}_{1166}$	-	-	-	+	-	-	-	-	-	-	-
ASN${}_{1167}$	-	-	-	+	-	-	-	-	-	-	-
ILE${}_{1130}$	+	-	-	-	-	-	-	-	-	-	-
ASN${}_{1171}$	-	-	-	-	-	-	-	-	-	-	-

## Supplementary Materials

The following are available online. Table S1: Comparison of the ensemble-docking results to the individual ones (a ligand against its own PDB-related structure), Table S2: most used c-Met inhibitors as pointed by SelleckChem and AdooQ Biosciences.

## Abbreviations

The following abbreviations are used in this manuscript:

DFG	aspartate-phenylalanine-glycine
MD	Molecular dynamics
RMSD	Root mean square deviation
