# Supplementary material for: Large-Scale Virtual Screening Against the MET Kinase Domain Identifies a New Putative Inhibitor Type
Source: Molecules. 2020 Feb 19;25(4):938. doi: 10.3390/molecules25040938 (PMC7070486; doi:10.3390/molecules25040938)
Supplement: Supplementary file 1 [file molecules-25-00938-s001.pdf]

**Supplementary Table I**: comparison of the ensemble-docking results to the individual ones (a ligand against its own PDB-related structure). In red the ones for which the preferred ensemble target is better than the PDB-related one (this correspond to a better score obtained)

| N° | Ligand PDB ID | Original PDB target | Docking score in the original PDB target | Preferred PDB target in the Ensemble docking | Docking score in the ensemble docking |
|---|---|---|---|---|---|
| 1 | KSA | MD_1R0P | 82 | MD_1R0P | 81 |
| 2 | AM7 | MD_2RFN | 101 | MD_2RFN | 102 |
| 3 | AM8 | MD_2RFS | 88 | MD_2RFS | 86 |
| 4 | CKK | MD_3C1X | 105 | MD_3C1X | 105 |
| 5 | LKG | MD_3CCN | 80 | MD_3CCN | 76 |
| 6 | L5G | MD_3CD8 | 85 | MD_3CD8 | 82 |
| 7 | 1FN | MD_3CE3 | 95 | MD_3CE3 | 95 |
| 8 | 319 | MD_3CTH | 92 | MD_3CTH | 90 |
| 9 | 320 | MD_3CTJ | 89 | MD_3CTH | 92 |
| 10 | IHX | MD_3F66 | 82 | MD_3F66 | 81 |
| 11 | 3F82 | MD_3F82 | 99 | MD_3F82 | 98 |
| 12 | 3EFJ | MD_3EFJ | 101 | MD_3EFJ | 102 |
| 13 | MT4 | MD_3EFK | 96 | MD_2RFN | 99 |
| 14 | ZZY | MD_2WD1 | 80 | MD_2WD1 | 78 |
| 15 | ATP | MD_3DKC | 106 | MD_3DKC | 102 |
| 16 | SX8 | MD_3DKF | 82 | MD_3CCN | 84 |
| 17 | VGH | MD_2WGJ | 82 | MD_2WGJ | 82 |
| 18 | DFQ | MD_3A4P | 81 | MD_3A4P | 79 |
| 19 | L8V | MD_3L8V | 82 | MD_2RFN | 85 |
| 20 | B2D | MD_3I5N | 83 | MD_3I5N | 86 |
| 21 | 88Z | MD_3LQ8 | 100 | MB_3LQ8 | 99 |
| 99 | PFY | MD_2WKM | 88 | MB_2WKM | 88 |
| 23 | Q6W | MD_3Q6W | 90 | MD_3Q6W | 93 |
| 24 | 3QT | MD_3QTI | 85 | MD_3QTI | 84 |
| 25 | M97 | MD_3RHK | 83 | MD_3RHK | 83 |
| 26 | KRW | MD_3ZXZ | 82 | MD_3CCN | 87 |
| 27 | 0JL | MD_4DEI | 94 | MD_4DEI | 93 |
| 28 | OJ3 | MD_4GG5 | 79 | MD_4GG5 | 77 |
| 29 | L1X | MD_4EEV | 106 | MD_3F86 | 111 |
| 30 | DF6 | MD_3VW8 | 98 | MD_3VW8 | 98 |
| 31 | 1JC | MD_4IWD | 98 | MD_4IWD | 97 |
| 32 | 5TF | MD_3ZCL | 75 | MD_3CCN | 76 |
| 33 | W97 | MD_3ZC5 | 79 | MD_2RFN | 81 |
| 34 | 6XE | MD_3ZBX | 78 | MD_3CCN | 82 |
| 35 | 1RU | MD_4KNB | 84 | MD_4KNB | 83 |
| 36 | DWF | MD_4MXC | 102 | MD_4MXC | 100 |
| 37 | 44X | MD_4XYF | 93 | MD_4XYF | 93 |
| 38 | 3E8 | MD_4R1V | 95 | MD_4R1V | 94 |
| 39 | 46G | MD_4XMO | 76 | MD_2WKM | 79 |
| 40 | 5B4 | MD_5DG5 | 95 | MD_5DG5 | 94 |
| 41 | 5T1 | MD_5EYD | 86 | MD_5EYD | 83 |
| 42 | 5QQ | MD_5EOB | 80 | MD_3CCN | 84 |
| 43 | 5SZ | MD_5EYC | 80 | MD_5EYC | 80 |
| 44 | 84P | MD_5UAF | | MD_5UAF | |
| 45 | 66L | 5HTI | 94 | MD_3F66 | 93 |

**Supplementary table II**: most used c-Met inhibitors as pointed by SelleckChem (www.selleckchem.com/c-Met.html) and AdooQ Biosciences (www.adooq.com/met.html)

| compound names | PubChem IDs | Tanimoto |
|---|---|---|
| Crizotinib | 11626560 | 0.31 |
| Cabozantinib | 25102847 | 0.24 |
| Foretinib | 42642645 | 0.29 |
| PHA-665752 | 10461815 | 0.39 |
| SU11274 | 9549297 | 0.38 |
| JNJ-38877618 | 57654476 | 0.27 |
| Glumetinib | 117797905 | 0.30 |
| Altiratinib | 54576299 | 0.27 |
| BMS-777607 | 24794418 | 0.31 |
| Tivantinib | 11494412 | 0.31 |
| JNJ-38877605 | 46911863 | 0.28 |
| PF-04217903 | 17754438 | 0.30 |
| Capmatinib | 25145656 | 0.31 |
| BMS-754807 | 24785538 | 0.33 |
| BMS-794833 | 44155856 | 0.32 |
| AMG-208 | 24864821 | 0.29 |
| MK-2461 | 44137946 | 0.29 |
| Golvatinib | 16118392 | 0.29 |
| AMG-458 | 24764449 | 0.34 |
| NVP-BVU972 | 44206063 | 0.26 |
| Tepotinib | 25171648 | 0.29 |
| AMG | 44181686 | 0.34 |
| Merestinib | 44603533 | 0.33 |
| S49076 | 49809909 | 0.36 |
| NPS-1034 | 46194178 | 0.35 |
| Savolitinib | 68289010 | 0.29 |
| SAR-125844 | 25182860 | 0.35 |
| SGX-523 | 24779724 | 0.26 |
| Glesatinib | 24901704 | 0.36 |
| BMS-817378 | 44137813 | 0.34 |
| MK-8033 | 45142457 | 0.28 |
| norcantharidin | 93004 | 0.12 |
